# Characterization of Protein–Protein Interfaces through a Protein Contact Network Approach

**DOI:** 10.3389/fbioe.2015.00170

**Published:** 2015-10-30

**Authors:** Luisa Di Paola, Chiara Bianca Maria Platania, Gabriele Oliva, Roberto Setola, Federica Pascucci, Alessandro Giuliani

**Affiliations:** ^1^Facoltà Dipartimentale di Ingegneria, Università Campus Bio-Medico di Roma, Rome, Italy; ^2^Department of Biomedical and Biotechnological Sciences, University of Catania, Catania, Italy; ^3^Dipartimento di Informatica e Automazione, Università degli studi Roma Tre, Rome, Italy; ^4^Dipartimento di Ambiente e Connessa Prevenzione Primaria, Istituto Superiore di Sanità, Rome, Italy

**Keywords:** anthrax toxin, protein–protein interactions, protein contact networks, network resilience

## Abstract

Anthrax toxin comprises three different proteins, jointly acting to exert toxic activity: a non-toxic protective agent (PA), toxic edema factor (EF), and lethal factor (LF). Binding of PA to anthrax receptors promotes oligomerization of PA, binding of EF and LF, and then endocytosis of the complex. Homomeric forms of PA, complexes of PA bound to LF and to the endogenous receptor capillary morphogenesis gene 2 (CMG2) were analyzed. In this work, we characterized protein–protein interfaces (PPIs) and identified key residues at PPIs of complexes, by means of a protein contact network (PCN) approach. Flexibility and global and local topological properties of each PCN were computed. The vulnerability of each PCN was calculated using different node removal strategies, with reference to specific PCN topological descriptors, such as participation coefficient, contact order, and degree. The participation coefficient *P*, the topological descriptor of the node’s ability to intervene in protein inter-module communication, was the key descriptor of PCN vulnerability of all structures. High *P* residues were localized both at PPIs and other regions of complexes, so that we argued an allosteric mechanism in protein–protein interactions. The identification of residues, with key role in the stability of PPIs, has a huge potential in the development of new drugs, which would be designed to target not only PPIs but also residues localized in allosteric regions of supramolecular complexes.

## Introduction

Protein–protein interactions play a key role in the biological signaling pathways; however, they were considered difficult to study in terms of their chemo-physical and structural properties. For a long time, scientists simply relied on the functional properties of protein complexes, disregarding the physical details of protein–protein interactions. This restriction had drastically limited the therapeutic interventions impinging on such interactions. This situation changed because of increased structural information; therefore, drugs specifically designed to target protein–protein interactions have reached the market (Arkin et al., 2014).

Once key residues (hot-spot residues) at protein–protein interfaces (PPIs) are identified, PPIs become challenging pharmacological targets with a huge potential in structure-based drug design (Wells and McClendon, 2007). Hot-spot residues largely contribute to the protein–protein binding energy and could be spread over wider “cold regions,” which comprise residues slightly contributing to complex stability (Clackson and Wells, 1995). Hot-spot residues are experimentally identified by mutating residues to alanine: hot spots are those residues that, if mutated, lead to a change in protein–protein binding energy >2.0 kcal/mol (Thorn and Bogan, 2001).

Experimental methods are generally expensive and time-consuming, and predictive computational methods may largely reduce time and cost by guiding the mutagenesis of putative hot-spot residues (Morrow and Zhang, 2012). Computational methods for prediction of hot-spot residues include computational alanine scanning, molecular dynamics, and machine learning-based methods (Morrow and Zhang, 2012); however, often it is necessary to combine methods in order to reach successful predictions.

The computational approach based on protein contact networks (PCNs) has been recently applied to study protein complexes (Di Paola et al., 2015). Although PCN is a minimalist tool, it is an efficient way to study protein structure–function relationships (Di Paola et al., 2013; Cheng et al., 2015). PCN formalism considers protein structures as networks whose nodes are amino acid residues, and two nodes (residues) are in contact if their distance in the 3D structure is within 4–8 Å. PCN helps in elucidating key processes, such as folding (Plaxco et al., 2000) and ligand binding (De Ruvo et al., 2012). Additional methods are based on centrality metrics and graph-spectral clustering, which are based on network partition into clusters of highly interconnected residues, through the analysis of global properties of PCNs (Brinda and Vishveshwara, 2005). PCN is also effective in the localization of hot-spot residues, by means of identification of highly connected hubs (residues) and clusters of nodes (residues), which corresponds to evolutionary conserved residues (Karain and Qaraeen, 2015). Furthermore, analysis of molecular dynamics, based on elastic network models, revealed that hot-spot residues show a moderate-high flexibility, which accounts for conformational accommodation upon binding (Chennubhotla et al., 2005).

Indeed, PCN approach has a high potential of applicability to the study of protein structures by means of simplified representations, e.g., networks and cluster maps. Therefore, we hereby propose an approach that links topology of PPIs to complex stability through the PCN formalism. We applied this method to complexes of protective agent (PA) bounded with lethal factor (LF) and CMG2. Furthermore, we analyzed monomeric and octameric PA. The analysis of PPIs for these complexes will open up the possibility to develop anthrax antidotes and possibly new anti-angiogenic agents.

The attention on anthrax toxicity raised when, in 2001, 22 people were exposed to mail envelopes contaminated by anthrax spores (Jernigan et al., 2001, 2002; Goel, 2015). The possibility of anthrax diffusion induced a widespread terror in the population, calling for huge attention from public authorities (Wein et al., 2003). Anthrax could be easily spread as bio-weapon, because of its low cost and high stability; moreover, anthrax spores can cause different forms of infection with a high mortality rate.

The molecular machinery behind the *Bacillus anthracis* toxicity relies on a trimeric protein complex (Young and Collier, 2007), which is composed of PA, LF, and edema factor (EF). Anthrax exerts its toxicity through the following steps: PA binding to extracellular domain of anthrax receptors (ANTXRs), PA oligomerization, binding of EF and LF, and endocytosis. EF and LF translocation through the PA pre-pore is promoted by low pH in the endosome (Young and Collier, 2007). So far, two ANTXRs have been cloned: the tumor endothelial marker 8 or anthrax receptor 1 (TEM8/ANTXR1) and the CMG2, also named as ANTXR2.

Toxicity of PA is mainly related to the activation of CMG2 receptor, because of its wider expression and higher affinity for PA compared to the TEM8 receptor. A key residue, leucine 56 in TEM8 mutated into alanine in CMG2, seems to influence PA affinity for ANTXR1 receptor (Fu et al., 2010). Indeed, the designing of drugs, which target PA/LF and PA/ANTXRs interfaces, could represent a key step in the development of an anthrax antidote. Furthermore, TEM8 and CMG2 receptors play a role in epithelial and endothelial cell functions, so that mutations of TEM8 and CMG2 lead to very rare diseases, whose pathological mechanism is still largely unknown (Deuquet et al., 2011). TEM8 is involved in the regulation of expression of vascular endothelial growth factor receptors (VEGFRs), playing a role in angiogenesis that, in turn, is detrimental in cancer progression (Deuquet et al., 2011). CMG2 is involved in the regulation cytoskeleton structure and might have a role in cancer spreading (Cryan and Rogers, 2011). The physiological functions of ANTXRs suggest that drugs targeting them would have a therapeutic potential for diseases where angiogenesis is detrimental (i.e., cancer and retinal neovascular diseases) (Cryan and Rogers, 2011).

The computational approach hereby presented aims at characterizing the homomeric and heteromeric interactions of PA. A PCN method was applied to crystal structures of the aforementioned complexes and was successful in finding “hot-spot” residues.

Analysis was focused on both global network stability (e.g., graph energy, flexibility, and robustness) and local features (e.g., participation coefficient, centrality, and degree). PCN approach has been further applied to evaluate complex stability, inferred from network resilience, or vulnerability (Oliva et al., 2013). The participation coefficient *P* was the topological parameter that mostly affected the PCNs’ vulnerability of all structures; meaning that, residues important for the protein–protein interactions are also involved in the inter-module communication. Inter-module communication is crucial for either allosteric mechanisms or cooperative events, which in turn play a key role in supramolecular interactions (Keskin et al., 2005). Therefore, identification of high *P* residues will help the rational drug design of molecules targeting supramolecular (protein–protein) interactions of anthrax complexes.

## Materials and Methods

### Protein Data Set

A series of X-ray structures were analyzed (Table 1). Structures are indexed with their own Protein Data Bank (PDB) code. The data set included monomeric and multimeric forms of PA, PA bound to LF (PDB: 3KWV), and to human receptor CMG2 (PDB: 1T6B).

**Table 1:** **Protein data set**.

PDB code	Description	Reference
1ACC	Anthrax toxin protective antigen (PA)	Petosa et al. (1997)
3Q8A	Anthrax PA wild type (pH 5.5)	Rajapaksha et al. (2012)
3Q8B	Anthrax PA wild type (pH 9.0)	Rajapaksha et al. (2012)
3KWV	Anthrax PA–PA–lethal factor	Feld et al. (2010)
3TEW	Crystal structure of PA	Feld et al. (2012)
1T6B	PA–CMG2	Santelli et al. (2004)
3HVD	PA octamer	Kintzer et al. (2009)

### Protein Contact Network

In PCN methodology, the protein structure is considered as a *graph* G = {*V*, ε}, where the set *V* includes the nodes *v*_1_…, *v*_n_ (i.e., the amino acid residues) and ε is the set of links (*v*_i_, *v*_j_), the link describes a specific relationship between i-th and j-th nodes. Topological descriptors of a PCN quantitatively describe the network that corresponds to a given protein structure (Csermely et al., 2013).

Protein contact networks were built on the base of three dimensional coordinates of α-carbons exported from PDB files (Table 1). Nodes of the network correspond to residues connected by links if the Euclidean distance between α-carbons is in the range of 4–8 Å; this range has been chosen because it includes only significant non-covalent intra-molecular interactions (da Silveira et al., 2009). PCN building is based only on the adjacency binary matrix **A**, whose rows and columns list residues ordered on the base of the protein primary sequence. A generic element *A*_ij_ of the adjacency matrix **A** is set to 1 if i-th and j-th nodes are connected by a link, otherwise *A*_ij_ is set to 0.

Protein structures, translated into a PCN, were then described in terms of (Csermely et al., 2013) (Table 2):

- local network descriptors that describe single residues properties (Table 2, upper part);
- global network descriptors that correspond to properties of the whole structure (average on the whole graph) (Table 2, lower part).

**Table 2:** **Topological descriptors**.

Variable	Description
**LOCAL**	
Degree	Node degree *k*_i_ is defined as the number of links each node forms:
	$k_{\text{i}}=\sum_{\text{j}}A_{\text{ij}}$
Clustering coefficient	The clustering coefficient *C*_i_ computes the number of triangles in a network where a node is involved
Contact order	Contact order ord_i_ computes for each node the average range (distance in sequence of adjacent nodes) of its contacts (Oliva et al., 2013):
	$\text{or}{\text{d}}_{\text{i}}=\frac{\sum_{\text{j}\neq \text{i}}|\text{i}-\text{j}|A_{\text{ij}}}{k_{\text{i}}}$
**GLOBAL**	
Average degree	The average degree $\bar{k}$ is the average value of node degree *k*_i_
Average clustering coefficient	The average clustering coefficient $\bar{C}$ is the average value of node clustering coefficient *C*_i_
Average shortest path	The average shortest path *asp* is the average value of the shortest paths matrix *SP* over all pair of residues
Graph energy	The graph energy *E* is defined as the sum of the adjacency matrix eigenvalues in module
Graph energy of complexes	The graph energy of complex formation *E*_C_ is computed as difference between the graph energy *E* computed for the whole complex and the corresponding value for single interacting chains
Inter-chain number of contacts	The inter-chain number of contacts *IC* represents the number of residues in pairwise contacts between different chains

*Local (single residue) and global (whole structure) descriptors of the topology of protein contact networks*.

Additionally, we computed two metrics of centrality:

- betweenness centrality: the number of shortest paths passing through each node. Nodes characterized by high betweenness centrality are crucial for signal transmission and, if placed in the PPI, likely responsible for inter-chain communication;
- closeness centrality: the sum of distances (shortest paths) of each node from other nodes. Indeed, high closeness centrality nodes are connected through few links to any other node of the network.

Furthermore, we applied a spectral clustering procedure in order to divide networks into clusters of nodes (Tasdighian et al., 2014). This procedure is based on eigenvalue decomposition. First of all, Laplacian matrix **L** of PCN is defined as the difference between the adjacency matrix **A** and the degree matrix **D**, which is a diagonal matrix, whose generic element *D*_ii_ is the i-th node degree. The eigenvalue decomposition is applied to **L**; therefore, eigenvectors are ordered according to the descending order of the corresponding eigenvalue. The reference for the spectral clustering decomposition is the second minor eigenvalue **v_2_**. The whole range of **v_2_** values is divided into as many sub-ranges as the number of clusters (given as input); therefore, a node is placed in a cluster according to which sub-range its **v_2_** value belongs.

The relevance of this approach, to study the structural basis of allosteric regions, was previously reported (De Ruvo et al., 2012; Di Paola and Giuliani, 2015). Once the PCN is partitioned into clusters, each node (residue) can be characterized in terms of its propensity to form inter-cluster or intra-cluster connections, by means of two descriptors:

- the participation coefficient *P*, defined as:
  (1) $$P_{\text{i}}=1-{\left(\frac{k_{\text{si}}}{k_{\text{i}}}\right)}^{2}$$
  *k*_si_ is the node degree in its own cluster. *P* measures the inter-cluster connectivity of nodes. *P* has been found to shift from non-null to null values in regions close to an allosteric site (De Ruvo et al., 2012);
- *z*-score of intra-cluster connectivity, defined as:
  (2) $$z_{\text{i}}=\frac{k_{\text{i}}-{\bar{k}}_{\text{si}}}{\sigma_{\text{si}}}$$
  ${\bar{k}}_{\text{si}}$ and σ_si_ are, respectively, the average and the SD of degree in the cluster to which the i-th node belongs. Therefore, *z*-score describes the propensity of a node to establish links with nodes belonging to its own cluster.

According to *P* and *z* values, nodes can be classified into seven categories, which cover specific regions in the *P*–*z* plane. This representation, called Guimerà–Amaral cartography (Guimerà and Nunes Amaral, 2005), characterizes the topological role of nodes in the PCN, according to their function in signal transmission across the network (Cumbo et al., 2014). The methodology is based on an evolution of the degree concept, which accounts for the ability of the nodes to link different regions of the network. This perspective is particularly well suited for PCNs, which are characterized by a strong small-world character; average shortest path in small-world networks scales logarithmically with the total number of vertices due to long-range contacts between residues far in sequence (Watts and Strogatz, 1998; Atilgan et al., 2004; Paci et al., 2012). *P*–*z* maps (representation of nodes distribution on the *P*–*z* planes) show a peculiar shape, referred to as “dentist’s chair,” which represents the PCNs of the folded protein structures. The “dentist’s chair” shape is conserved for a large number of structures, and reveals a common architecture of protein folds regardless of protein function and size (Krishnan et al., 2007). Clustering partition, through a clustering color map, was hereby represented as a 2D map clusters of residues (Tasdighian et al., 2014), where each cluster is represented by a different color and projections of clusters correspond to long-range interactions. This representation helps in characterizing the match between clusters and chains, or protein domains.

### Flexibility and Vulnerability Assessment

The flexibility index *f* of a network with *c* degrees of freedom is defined as (Thorpe and Kuhn, 2000):

(3) $$f=\frac{c-6}{c}$$

because the maximum number of degrees of freedom in a rigid network in 3D is 6.

The *f* index was calculated accordingly to Eq. 3 (Table 3) and degrees of freedom of PCNs were calculated accordingly to the method of Zelazo et al. (2012). The vulnerability of PCNs was computed in terms of size reduction of the largest giant component (LGC) of each PCN (Holme et al., 2002; Oliva et al., 2013); this reduction of size is defined also as “biggest fall.” The LGC of the graph is the set of connected nodes with the maximum number of nodes. The reduction profile of the size of LGC depends on the specific strategy of nodes removal: nodes with decreasing or increasing values of a given network descriptor are removed. A huge fall in LGC size, due to a small fraction of removed nodes, identifies a network vulnerable to a given removal strategy, whereas the network is robust if a smooth and regular degradation is measured.

**Table 3:** **Flexibility analysis results**.

PDB code	Degrees of freedom	Flexibility *f*
1ACC	36	0.83
3Q8A	40	0.85
3Q8B	38	0.84
3TEW	46	0.87
3KWV	30	0.80
3HVD	21	0.71
1T6B	26	0.77

The synthetic index of vulnerability, the Degradation Index *DI*, (Oliva et al., 2013), is defined as the normalized metric of the effectiveness of different removal strategies:

(4) $$\text{DI}=\frac{\text{Difference in giant component size}}{\text{Fraction of removed nodes}}$$

The index is computed at the first discontinuity in size of the giant component and accounts for the magnitude of the fall of the LGC and for the fraction of removed nodes that generated the fall.

### Figures, Graphs, and Statistics

Figures of the protein structures were obtained via PyMOL Molecular Graphics System, Version 1.7.4. Values of the molecular descriptors of residues were mapped in each structure by adding the column corresponding to the *b*-factor in the PDB file. Graphs and statistics were obtained with Matlab 2014a environment.

## Results

### Protein Contact Networks General Properties

Global topological descriptors of PCNs (Table 4) of monomeric forms were not markedly different from descriptors of complexes; this result suggested that PPIs would not influence the overall topology of PCNs. The parameter that best described PPIs is the inter-chain energy of the graph *E*_C_. The percentage of residues involved in inter-chain contacts (*IC)* varied from 8.7% (PDB: 3KWV) to 4.4% (PDB: 1T6B), whereas the inter-chain energy *E*_C_ normalized per inter-chain contacts (*E*_C_*/IC*) substantially did not vary; meaning that *E*_C_ only depends on the number of residues in the PPIs and not on their nature (contact length and type). Greater *E*_C_ values described complexes bearing homomeric PA interactions (PDB: 3HVD and 3KWV).

**Table 4:** **Topological and structural descriptors**.

	*N*	$\bar{\text{k}}$	$\bar{\text{C}}$	$\bar{\text{SP}}$	*E*	ORD	E_C_	IC (%)	*E*_c_/*IC*	*R*_G_	MDF	ε
1ACC	665	7.91	0.30	7.77	1462.1	302.18	–	–	–	16.11	1.89	0.68
3Q8A	675	7.87	0.30	7.81	1476.8	288.91	–	–	–	16.09	1.90	0.64
3Q8B	676	7.95	0.30	7.77	1486	298.71	–	–	–	16.12	1.86	0.64
3TEW	717	8.03	0.29	7.90	1570.8	306.17	–	–	–	16.42	1,56	0.63
3KWV	1275	7.71	0.29	9.86	2767.8	337.64	38.45	111 (8.7)	0.35	18.89	1.80	0.75
3HVD	1041	7.98	0.30	8.73	2305.7	349.41	23.4	69 (6.6)	0.34	16.25	2.40	0.73
1T6B	846	8.08	0.29	9.10	1876.2	304.52	14.33	37 (4.4)	0.39	19.25	0.87	0.78

### “Hot-Spots” Recognition by Mesoscopic Topological Descriptors

**Figure 1:**
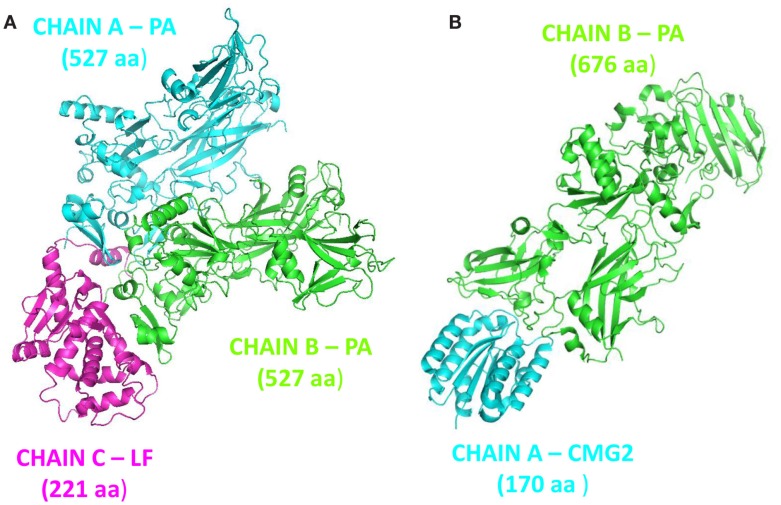
**Complex structures: (A) PA–PA–LF trimeric complex (PDB: 3KWV); (B) dimeric complex PA–CMG2 (PDB: 1T6B)**.

Protective agent bound to LF (PDB: 3KWV) and to receptor CMG2 (PDB: 1T6B) was analyzed (Figure 1), because drugs targeting key residues of such complexes may be developed as anthrax antidotes or novel anti-angiogenic molecules.

**Figure 2:**
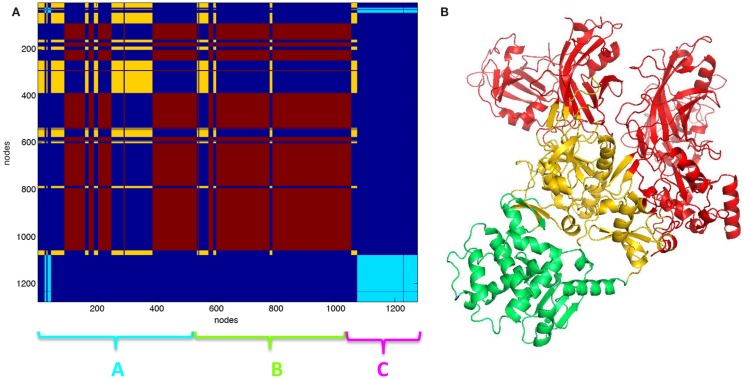
**Clustering partition of PA–PA–LF complex: (A) clustering color map, the length of the three chains is reported for reference; (B) cartoon representation of clusters the green cartoon corresponds to the light blue cluster in (A)**. The lethal factor (LF) is highlighted by the empty circle.

The clustering of PCN of 3KWV, accordingly to graph-spectral method, was carried out (Figure 2). The protein network was divided into three clusters. The clustering helped for the identification of protein modules of the complex. The two chains of PA were not recognized as two different clusters; otherwise, each PA chain was divided into two functional modules: the red cluster (Figure 2A) is the protein module far from PPI (red cartoon Figure 2B), the yellow cluster (Figure 2A) is the protein module near the PPI (yellow cartoon Figure 2B) and in contact to the LF (green cartoon Figure 2B). The red and yellow clusters were substantially intermingled (Figure 2A).

**Figure 3:**
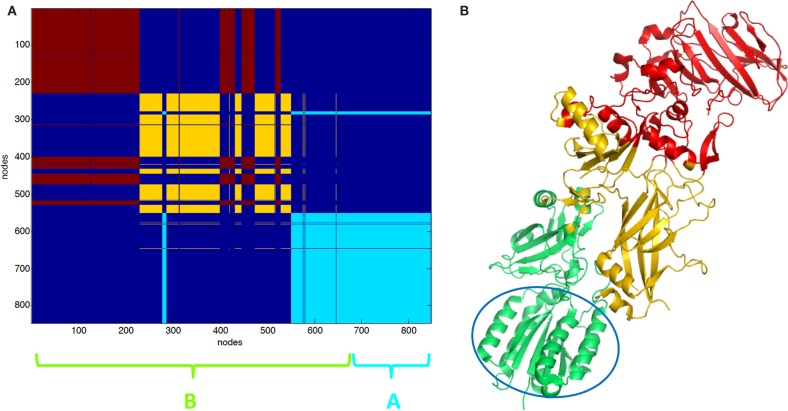
**Clustering partition of PA–CMG2 complex: (A) clustering color map, the length of the three chains is reported for reference; (B) cartoon representation of clusters the green cartoon corresponds to the light blue cluster in (A)**. The CMG2 receptor is highlighted by the empty circle.

Partition in three clusters of PA–CMG2 complex (1T6B) was also carried out. In 1T6B, a single PA chain is bound to CMG2. PA is divided into two intermingled functional domains, such as the yellow and the red clusters (Figures 3A,B). The light blue (Figure 3A) cluster includes the CGM2 and a domain of PA (cartoon Figure 3B).

**Figure 4:**
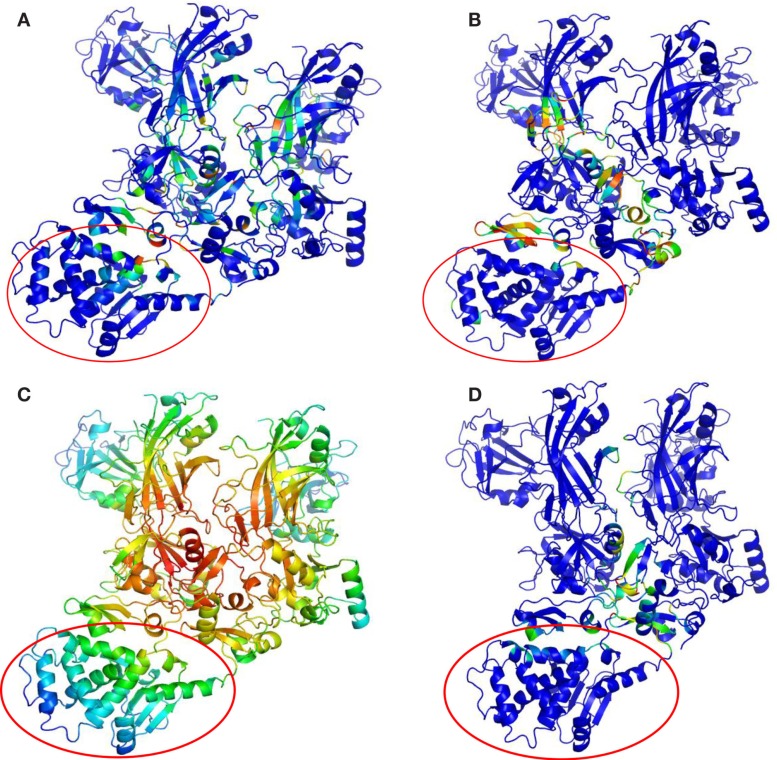
**Maps of PCN descriptors on protein structures: complex PA–PA–LF (PDB: 3KWV): (A) betweenness centrality; (B) participation coefficient *P*; (C) closeness centrality; (D) inter-chain degree**. The lethal factor (LF) is highlighted by the empty circle.

**Figure 5:**
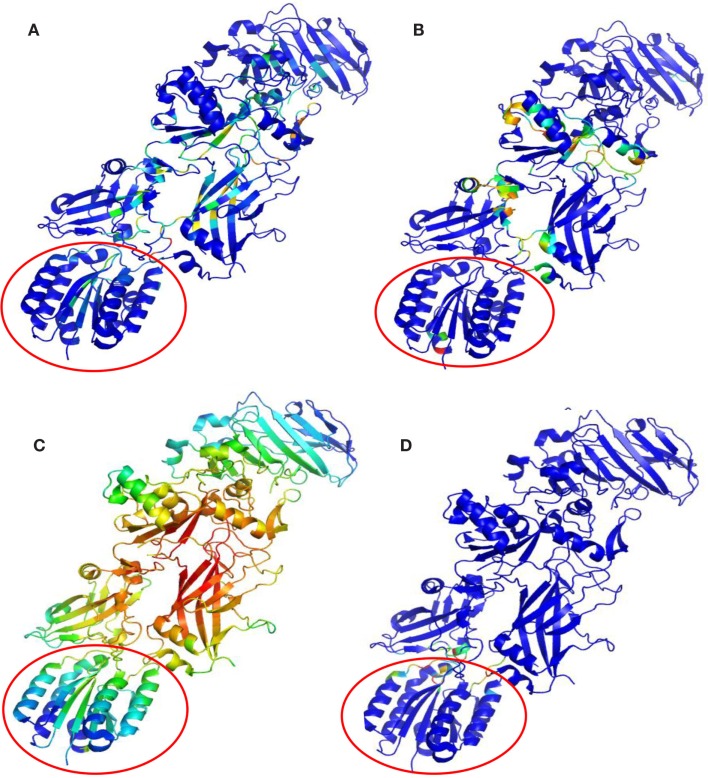
**Maps of PCN descriptors on protein structures: complex PA–CGM2 (PDB: 1T6B): (A) betweenness centrality; (B) participation coefficient *P*; (C) closeness centrality; (D) inter-chain degree**. The CMG2 receptor is highlighted by the empty circle.

Key PCN descriptors were mapped in the molecular representation of PA–PA–LF (PDB: 3KWV) and PA–CGM2 (PDB: 1T6B) (Figures 4 and 5). In 3KWV, the residues with highest closeness centrality (Figures 4C and 5C) belong to the center of mass of the complex, where the residues have the lowest flexibility. In fact, high closeness corresponds to high rigidity of the residue. Conversely, betweenness centrality (Figures 4A and 5A) and participation coefficient *P* (Figures 4B and 5B) identify residues at the PPIs, which would be good candidates as hot-spot residues. Betweenness centrality and participation coefficient *P* maps also highlighted residues far from interfaces, in regions with possible allosteric function. Finally, the inter-chain degree (Figure 4D) identified the residues in PPIs easily. The role of inter-chain degree in the identification of residues at PPIs (PA–LF, PA–PA, and PA–CMG2) confirmed the results about the role of *E*_c_ in description of PPIs of complexes.

### Protein Contact Networks Rigidity and Vulnerability Analysis

All structures showed a flexibility index *f* (Table 3) >0.7, albeit with some differences. 3TEW was the most flexible (*f* = 0.8696), and 3HVD was the most rigid (*f* = 0.7143). Flexibility was high in monomeric forms of PA, and decreased with increasing number of interacting chains. In fact, flexibility was lowest in 3HVD where eight chains of PA are interacting. The comparison of flexibility of PA–PA–LF (PDB: 3KWV) and PA–CMG2 (PDB: 1T6B) revealed that the number of PA chains did not decrease the flexibility of 3KWV; although, the difference in values was not high.

**Figure 6:**
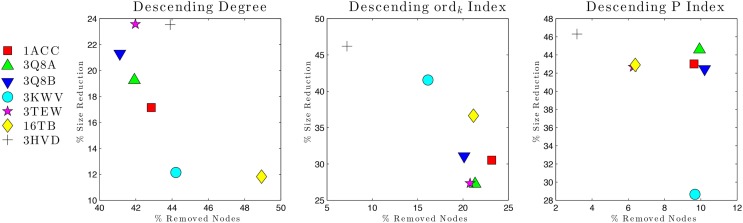
**Network vulnerability upon nodes removal strategies**.

**Figure 7:**
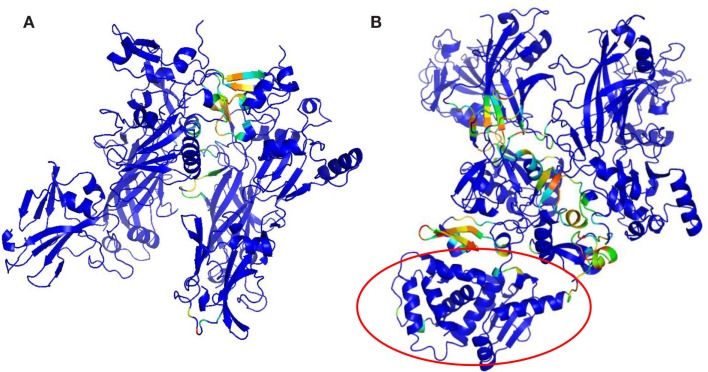
**P maps on protein structures: (A) 3HVD, octamer of PA; (B) 3KWV, PA–PA–LF complex**.

Vulnerability analysis was done by removing nodes with decreasing values of *P*, *contact order*, and *degree* (Table 5; Figures 6 and 7). The *P* removal strategy significantly influenced the dimension of LGC, i.e., the biggest fall of LGC is associated to the lowest percentage of removed nodes. In all structures, the participation coefficient *P* was the parameter with the highest influence on PCN robustness. The most vulnerable structure was the octameric PA (PDB: 3HVD), which showed the biggest fall with 3% of removed nodes. The PA–PA–LF complex was less vulnerable than PA–CGM2; meaning that PA–PA interaction were not crucial in the participation coefficient *P* vulnerability strategy, at least for heteromeric interactions. The *contact order* removal strategy was substantially detrimental for network robustness of the octameric structure of PA (PDB: 3HVD) (7% of removed nodes) and of PA–PA–LF complex (PDB: 3KWV) (16% of removed nodes); in this case, PA–PA interaction influenced the robustness of PCNs. Finally, the *degree* removal strategy did not influence the robustness of all PCNs. The influence of *P* parameter was different in homomeric (PDB: 3HVD) and heteromeric complexes (PDB: 3KWV). Map of residues with high *P* values in the homomeric (Figure 7A) and heteromeric (Figure 7B) complexes of PA is reported in Figure 7. High *P* residues were at PA–PA interfaces; however, high *P* values in 3KWV were localized also at PA–LF interface.

**Table 5:** **Vulnerability analysis**.

PDB code	Biggest fall	% Removed nodes	DI index
**DESCENDING P**			
1ACC	286 (43.01%)	9.62	4.47
3Q8A	301 (44.59%)	9.93	4.49
3Q8B	287 (42.46%)	10.21	4.16
3TEW	306 (42.68%)	6.28	6.8
3KWV	151 (28.65%)	9.68	2.96
3HVD	482 (46.30%)	3.17	14.61
1T6B	363 (42.91%)	6.38	6.72
**DESCENDING CONTACT ORDER**			
1ACC	203 (30.53%)	23.16	1.32
3Q8A	184 (27.26%)	21.33	1.28
3Q8B	210 (31.07%)	20.12	1.54
3TEW	196 (27.34%)	20.78	1.32
3KWV	219 (41.56%)	16.13	2.58
3HVD	481 (46.21%)	7.20	6.41
1T6B	310 (36.64%)	21.16	1.73
**DESCENDING DEGREE**			
1ACC	114 (17.14%)	42.86	0.40
3Q8A	130 (19.26%)	41.93	0.46
3Q8B	144 (21.30%)	41.12	0.52
3TEW	169 (23.57%)	41.98	0.56
3KWV	64 (12.14%)	44.21	0.27
3HVD	245 (23.54%)	43.90	0.54
1T6B	100 (11.82%)	48.94	0.244

## Discussion

Crystal structures of unbound anthrax PA, octameric form of PA, PA–PA–LF, and PA–CMG2 complexes were analyzed using a PCN approach. This methodology was used in order to identify residues (hot-spots) that influence significantly the stability of complexes.

Protein–protein interface properties were largely independent from global topology descriptor of the systems. For this reason, local (single residue) properties of PCNs were analyzed in detail.

Local topological descriptors that better described PPIs were the betweenness centrality, the participation coefficient *P*, and the inter-chain degree. Furthermore, hot-spot residues with the highest betweenness centrality and the participation coefficient were localized in regions with allosteric functions. This result complies with previous works, hot-spot residues are not necessarily placed PPIs, and they can be localized in other regions of the complex acting through allosteric mechanisms (Reynolds et al., 2011). Residues far from PPIs (Reynolds et al., 2011) modulate the small-world properties of PCNs explaining the allosteric nature of PPIs (Brinda et al., 2002).

Thus, the general wiring architecture of PCNs provides a mesoscopic framework (clustering, small-world nature) influencing the “hot-spot” character of residues. This result is in accordance with the previous findings about the modular feature of protein structures (Di Paola et al., 2012; Tasdighian et al., 2014).

Analysis of global PCN vulnerability identified which residues have a crucial role in the stability of anthrax complexes. The PCN vulnerability assessment showed that high *P* residues played a key role in the network stability of all analyzed protein structures. Furthermore, participation coefficient *P* and the contact order described residues likely to be involved in cooperative binding. In the PA octamer (3HVD), the most vulnerable structure, high *P* residues were mostly localized at PPIs; this means that inter-module communication guide cooperative binding and influence PCN robustness. In the PA–PA–LF complex, high *P* residues were specifically at PA–LF and PA–PA interfaces and also in allosteric regions. Furthermore, PA–CMG2 complex was more vulnerable than PA–PA–LF based on P removal strategy. Thus, the participation coefficient *P* accounted for overall structural stability and high *P* residues were localized at PPIs and in allosteric regions. Furthermore, the participation coefficient *P* partially explained cooperative events. However, the parameter that mostly explained cooperative events was the *contact order*, because the most vulnerable structures were the octameric PA and the PA–PA–LF complexes, where there are homomeric PA–PA interactions. Interestingly, all analyzed structures were similarly resistant to node removal based on decreasing *degree*, which is known to be highly effective for scale-free networks (Holme et al., 2002). In conclusion, network vulnerability assessment identified high *P* residues, distributed both at PPIs and regions far from PPIs, as the most essential for global stability.

The identification of high *P* residues, crucial for supramolecular interactions, would have a perspective application as a guide for site-directed mutagenesis and other experimental approaches, aimed at functional characterization of PPIs. Furthermore, this approach will help medicinal chemists to identify cavities for molecular-docking studies, in order to design effective drugs targeting protein–protein interactions. Indeed, finding of high *P* residues will open additional scenarios to drug discovery applications. Drugs may not target PPIs, often scarcely accessible. Additionally, drugs would be designed to bind allosteric regions, influencing supramolecular interactions with efficacy comparable or higher than drugs targeting only protein–protein interfaces.

## Conflict of Interest Statement

The authors declare that the research was conducted in the absence of any commercial or financial relationships that could be construed as a potential conflict of interest.
